# Leo Schamroth: his contributions to clinical electrocardiography

**Published:** 2009-02

**Authors:** R Scott Millar

**Affiliations:** Department of Medicine, Cardiac Clinic, University of Cape Town, Observatory

## Abstract

**Summary:**

Leo Schamroth (1924–1988) was one of the best-known South Africans in the international medical community. His book, *An Introduction to Electrocardiography*, first published in 1957, was my introduction to the mysteries of the ECG. The first edition was only 90 pages and was a model of clarity and simplicity, with remarkable insight into the needs of a student new to the subject. It has been translated into Spanish, Italian, Greek, Turkish and Japanese, and is said to be the book most often stolen from medical libraries worldwide.^1^

Schamroth was a superb teacher, not only of the ECG, and will be remembered by generations of students who passed through his hands during his tenure at the Chris Hani-Baragwanath Hospital from 1956 to 1987, occupying the Chair of Medicine there from 1972. As a lecturer who combined unrivalled clarity with showmanship, he held his audiences, at home and all over the world, spellbound. However, it was his ability to wring insights from the most ordinary-appearing ECG, by painstaking analysis, that is his enduring legacy.

## Written legacy

In addition to eight books, he published over 200 articles, many of which were co-authored by junior doctors, who were presented with the opportunity of a virtually guaranteed publication. He wrote on all aspects of the ECG, bringing his unique combination of observation, analysis and clarity of exposition.

## The ECG

Schamroth’s foremost contribution was his book *An Introduction to Electrocardiography*, which ran to eight editions, the last of which was published posthumously and edited by his son, Colin. This book introduced generations of undergraduate and postgraduate students to the intimidating mysteries of the ECG, explaining this with the aid of simple illustrations and ladder diagrams. It was probably the most popular introduction to the ECG throughout the world.

His magnum opus, *The 12 Lead Electrocardiogram*, was published posthumously. Although his books are out of print, they are still in demand and are available through various internet providers, at relatively high prices.

## Arrhythmias

*The Disorders of Cardiac Rhythm* was enthusiastically received on its publication in 1971. Here he covered arrhythmias, on which he had been writing for years, with information on mechanisms and treatment, together with detailed discussion on illustrative cases.

***Sinus node dysfunction:*** Among other publications on sinus node function, is an elegant article on the recognition and features of Wenckebach sino-atrial block.^2^ This phenomenon is infrequently recognised and depends on careful analysis of P–P intervals preceding and following the sinus pause, as there is no direct recording of sinus node discharge on the ECG. The clue to the Wenckebach mechanism is that the P–P interval shortens before the missing P wave, just as the R–R interval shortens before the dropped beat in the Wenckebach atrio-ventricular block. This is due to the fact that, while the P–R interval continues to lengthen, the increment diminishes. The contribution of the P–R interval is therefore greatest for the first R–R interval of the sequence, and lessens thereafter.

***Bundle branch block and phasic aberrant ventricular conduction:*** Schamroth popularised the concept of phasic aberrant ventricular conduction, i.e. rate-related bundle branch block, emphasising that aberrantly conducted complexes may be mistaken for ventricular ectopics.^3^ This has, unfortunately, been misinterpreted by many practitioners to suggest that supraventricular tachycardia with aberration is a more likely cause of a wide QRS tachycardia than is ventricular tachycardia, which is not the case.

The concept of incomplete left bundle branch block (LBBB) had been demonstrated experimentally, but not clinically, until the 1964 article by Schamroth and Bradlow.^4^ Here they showed an ECG from a patient in which there was a gradual change from normal conduction to incomplete LBBB, as shown by the loss of the septal Q wave in V5, with gradual widening of the QRS until complete LBBB was present. Because this occurred in the same patient and within a short time, it validated the existence and features of incomplete LBBB for the first time.

In both of these articles, the mechanism of rate-related bundle branch block is clearly described and illustrated with diagrams. Schamroth emphasised the importance of different refractory periods in the two bundle branches and the effects of changes in the R–R interval on His-Purkinje refractoriness. The apparent paradox of aberration occurring when a premature supraventricular complex follows a longer R–R interval, whereas conduction is normal at the same prematurity if the preceding R–R is shorter, is accounted for by the bundle branch refractory period shortening in response to the shorter R–R.

***Atrioventricular (AV) conduction and heart block:*** Schamroth wrote articles on many aspects of atrio-ventricular conduction, including concealed conduction, the supernormal phase and Wedensky facilitation.

***Ventricular ectopy and parasystole:*** Schamroth was fascinated by ventricular ectopic rhythms.^5^ Much of his prodigious output was devoted to detailed analysis of bigeminy, parasystole and concealed ventricular extrasystoles. He was adept at detecting the far-from-obvious relationships between apparently unrelated ventricular complexes.

## Ischaemic heart disease

His books, articles and presentations covered all aspects of the subject, culminating in *The Electrocardiology of Coronary Artery Disease.* One of his most memorable lectures was on the diagnosis of myocardial infarction in the presence of LBBB, which helped to debunk the myth that it could not be done.

## The defence of Don Craib

William Hofmeyer (Don) Craib’s contribution to the theory and development of the ECG is discussed elsewhere in this issue. It was, however, Leo Schamroth who was responsible for resuscitating his reputation and highlighting his singular contribution to the science of electrocardiology. He did this in 1977 in the AJ Orenstein memorial lecture, titled ‘The trial of William Craib’, in which he took the part of the defence in a mock trial for alleged plagiarism of research results. This was based on Craib’s monograph to the Rockefeller Foundation, titled ‘In defence of honour’. It was a theatrical masterpiece, which received wide acclaim and resulted in Craib being elected to the Society of Scholars of Johns Hopkins University, which had failed to protect him when he was falsely accused of plagiarism and shunned by his peers.

## Schamroth’s sign

Schamroth had the misfortune to suffer from rheumatic valve disease and developed infective endocarditis. Typically, he used the opportunity to observe and describe a simple sign to confirm the presence of clubbing of the fingers, now known as Schamroth’s sign, in which the fingernails of each hand are placed against each other. If clubbing is present, the normal gap between the bases of the nails is filled in.^6^

## Medical significance

Schamroth’s contribution to the understanding of the ECG by students, non-specialists and physicians during his lifetime was immense. He communicated his enthusiasm and flair for unearthing the hidden meaning in tracings to all who came in contact with him. His influence extended worldwide through his books, articles and lectures and he elevated the ECG from a clinical tool to an intellectual delight. He built upon the traditions of the Chicago School of Electrocardiography and was in the same league as Richard Langendorf and Alfred Pick.

While he did not ignore the clinical significance and context of his many observations, his focus was on the detailed analysis of timing and associations of ECG events to reveal relationships that were hidden from the casual observer. He was slow to embrace the rapidly developing field of invasive cardiac electrophysiology, although it confirmed many of the brilliant deductions made by him and others, based on the surface ECG alone.

Most notably, he was reluctant to accept the concept of reentry as the basis of most sustained ventricular tachycardias, preferring the concept of focal ectopic tachycardias. This was based on his calculations that ventricular conduction velocity and refractory periods largely precluded re-entry, although he conceded that bundle branch re-entry could occur.^7^ Re-entry has been shown to be the mechanism of most clinically important tachycardias and forms the basis for much of the modern therapy, including pacing termination of ventricular tachycardia by implanted devices and ablation of arrhythmia substrates, including those in the left ventricle.

Schamroth concentrated much of his intellectual effort on the intricacies of ventricular ectopy, parasystole and the rule of bigeminy. This work still stands as a monument to rigorous analysis, but its clinical significance is somewhat limited.

His clear exposition of the phenomenon of phasic aberrant ventricular conduction is as valid today as it was then. It nevertheless still causes confusion and is sometimes enthusiastically over diagnosed by those less skilled in ECG interpretation, resulting in misdiagnosis of ventricular tachycardia as supraventricular.

## Conclusion

Leo Schamroth was a giant in the field of deductive electrocardiography and achieved international renown. He was part of a fertile group whose remarkable deductive skills laid the basis for much of our current knowledge on the ECG. His comment regarding Richard Langendorf, that his work entailed ‘pure, pristine, logical Aristotelian thought’^8^ applies equally to Schamroth himself. Modern electrophysiology, both clinical and experimental, has subsequently made huge strides, which have revolutionised our management of patients with cardiac arrhythmias and generated a large amount of information that has greatly improved our understanding of the 12-lead ECG.

Schamroth’s untimely death in 1988 came before some of the most dramatic developments in catheter ablation and the use of implantable cardioverter defibrillators. I’m sure that he would have continued to find much to stimulate his fertile brain and remind us how much could be discovered by his sleuth-like approach to the secrets hidden in the squiggles recorded from hearts over the course of more than a century since the discovery of the electrocardiogram.
